# Tuberculosis treatment outcomes among prisoners and general population in Zomba, Malawi

**DOI:** 10.1186/s12889-020-08841-z

**Published:** 2020-05-15

**Authors:** Victor Singano, Esther Kip, Wilson Ching’ani, Lawrence Chiwaula

**Affiliations:** 1 Dignitas International, Zomba, Malawi; 2 Mothers2Mothers, Lilongwe, Malawi; 3 Department of Mental Health, College of Medicine, Blantyre, Malawi; 4 Zomba District Health Office, Zomba, Malawi; 5 Prison Health Services, Zomba, Malawi

**Keywords:** Tuberculosis, Treatment outcome, Prisoners, General population

## Abstract

**Background:**

TB remains a major global health problem. It is particularly prevalent in prisons in sub-Saharan Africa due to overcrowding, malnutrition, high HIV prevalence and insufficient medical services. Prisoners have experienced worse TB treatment outcomes than the general population. The researchers investigated the TB treatment outcomes and predictors of unsuccessful treatment outcomesamong prisoners and the general population in Zomba, Malawi.

**Methods:**

We retrospectively reviewed TB registers of prisoners and the general population diagnosed with TB from January 2011 to December 2016 at Zomba Maximum Central Prison and Zomba Central Hospital, Malawi. The study used routinely collected data extracted from national, standardized TB treatment monitoring tools. Successful treatment outcome was classified as the total for cured and completed treatment while unsuccessful treatment outcome was classified as the total of deaths and treatment failures. We used descriptive statistics to compare the demographics and TB treatment parameters among prisoners and non – prisoners and computed multivariate analysis to predict the independent factors of unsuccessful treatment outcomes.

**Results:**

Of 1652 registered cases, 27% were prisoners (all males) and 72% were non-prisoners (58% males). The median age was 35 years (IQR: 29–42); 76% were Pulmonary TB cases (78% among prisoners vs 75% among general population); 83% were new TB cases (77% among prisoners vs 86% among general population); and 65% were HIV positive (50% among prisoners vs 71% among general population). Regarding treatment outcome, 1472 (89%) were cured and/or completed treatment (93% among prisoners vs 88% among general population), 2(0.2%) were treatment failures, 122 (8%) died (5% among prisoners vs 8% among general population) and 55 (3%) were not evaluated (1% among prisoners vs 4% among general population). Unsuccessful TB treatment outcomes were associated with age greater than 35 years (aOR = 0.68: 95% C.I: 0.58–0.80), Extra-Pulmonary TB (aOR = 1.69: 95% C.I: 1.08–2.63) andHIV positive status (aOR = 0.63: 95% C.I: 0.42–0.94).

**Conclusion:**

Maximum prisons provide a stable population that can be easily monitored throughout the course of TB treatment. Good TB treatment outcomes which are comparable to the general population can be achieved among Malawian prisoners despite the challenging prison conditions.

## Background

Tuberculosis (TB) remains a major global health problem. Globally in 2018, there were an estimated 10 million new TB cases of which 8.6% were among People living with HIV [1]. There were an estimated 1.4 million TB deaths worldwide with 17% (0.2 million deaths) resulting from TB disease among people living with HIV [1]. TB is the most common severe opportunistic infection associated with HIV in many resource-limited countries [2] with 68% of incident TB cases reported in the World Health Organization (WHO) South East Asia and African Regions [1]. TB is more common in prison populations than in the general populations [2] and HIV epidemic, poor living conditions, low socioeconomic status and poor general health, appears to have contributed directly to higher rates of TB within prisons [2]. Prisons therefore serve as reservoirs for TB infection, not only for inmates and staff but also for the general population as visitors come and go and released prisoners return to society [2]. A systematic review showed that 8.5 and 6.3% of TB in the general population in high- and middle/low income countries, respectively, was attributed to the TB in prisons [3].

In 2018, Malawi reported an estimated 15,000 TB case notifications (181 cases per 100,000 population) with a 48% HIV/TB co-infection rate (99% on ART {anti-retroviral therapy}) and 86% TB treatment success rate [1]. Malawi is one of the focus countries in the *End TB Strategy* era (2016–2035), as it is in the top 30 countries of high TB incidence among people living with HIV [1].

Data from the WHO epidemiological surveillance for TB treatment outcomes of 2017 cohort showed TSR of 76% in the WHO Region of the Americas(due to high levels of loss to follow up and missing data), 78% in the European Region (due to high rates of treatment failure and deaths, from high rates of MDR-TB),; 83% in South East Asia; and82% in sub – Saharan Africa [1]. In contrast, Prison settings have shown sub-optimal TB treatment success rates than the recommended 90% set by the *End TB Strategy*. TB treatment outcomes’ studies among prisoners showed TSR of 50, 45, 48% in Brazil(2011) [4], South Africa(2009) [5] and Uganda(2012, [6]), respectively. The poor TB TSR in prisons might compromise TB control and contribute to the development of Multi Drug Resistant Tuberculosis (MDR-TB), currently at a rate 7.7 per 100,000 population in the WHO African Region [1]. In Malawi (2014), the prevalence of MDR-TB was 0.4% among new cases and 4.8% among retreatment cases [7].

Standard TB treatment outcomesare known to be influenced by a number of factors including HIV co-infection, but the evidence on factors that are most influential for prisoners,is not known [2]..

While some studies found lower TB treatment success rates among TB/HIV patients [8–10], other reported comparable rates to those infected only with TB [11]. Since 2011 Dignitas International (DI) in conjunction with the Ministry of Health has been providing integrated HIV/TB services to Zomba Central Hospital and Zomba Central Prison in Zomba, Malawi. The Zomba Central Prison is overcrowded (houses approximately 2500 prisoners at any point in time for a facility meant for 800 prisoners). Despite the evidence on the higher TB cases in prisons than the general population, TB TSR are not disaggregated according to prisonsTB TSR serves as a proxy for the quality of TB control as a secondary prevention. Therefore,we investigated the TB treatment outcomes and predictors of unsuccessful treatment outcomes among prisoners and non – prisoners to audit the effectiveness of TB control in identifying gaps in the national treatment policy and practice in prisons to initiate evidence based practice. .

## Methods

### Study design and setting

A retrospective cross-sectional study was conducted at the Zomba Central Prison (ZCP) and Zomba Central Hospital (ZCH), Malawi. The sites were purposefully selected. Zomba Central Prison is the maximum security prison with the highest prison population in Malawi. On average, it houses approximately 2500 prisoners in a facility meant for 800 prisoners. Zomba Central Hospital is a tertiary semi - urban referral hospital (catchment area 3.1 million) for the South East Health Zone in Zomba, Malawi.

The data source was the National TB register where relevant clinical information for all patients diagnosed with active TB disease are monitored throughout the course of their treatment.

### Organization of Health and TB services in Malawi

Malawi follows a three tier health care system which is connected by a patient referral system. Primary care where the bulk of health care occurs consists of hospitals that provide out-patient services, holding wards and community based outreach services. Secondary level care is provided by district hospitals in 28 districts which provide the same basic services as the primary care facilities in addition to a few more, such as: radiology, ambulance, operating theatre and basic laboratory. The central hospitals (located in 4 urban areas) provide tertiary specialized services.

There is decentralized provision of TB and HIV services. TB diagnostic services include smear microscopy (widely available in primary care facilities) and GeneXpert MTB/RIF (district and central hospitals) on spot and morning sputum samples. The National TB Reference Laboratory provides high level diagnostic services, including solid and liquid culture and drug sensitivity testing. It is also responsible for quality assurance services to the peripheral laboratories (central and district hospitals). TB treatment follows Direct Observed Treatment Strategy (DOTS).

Malawi prisons are categorized into high and low facilities. The high volume facilities (central prisons) have onsite clinics that provide primary care services and basic TB diagnostic services (smear microscopy and gene-expert). Low volume prisons do not have onsite clinics but depends on referring patients to the nearest primary or secondary care facility. In the central prisons, prisoners are symptomatically screened for TB, Sexually Transmitted Infections (STIs) and HIV during their entry, stay and exit by prison wardens. Prisoners with cough submit spot and morning samples which are analyzed using either smear microscopy or GeneXpert MTB/RIF at the prison laboratory (current Malawi TB program guidelines require GeneXpert MTB/RIF as first test for sputum from prisoners). Prisoners suspected of smear-negative PTB or extra-pulmonary TB (EPTB) are referred to the nearest district or central hospital for further investigations. Dignitas International provided clinical officers and nurses to support direct service delivery in the prison clinic (due to the shortage of health workers in the prison), and trained and supervised the prison wardens in DOTS.

TB services data is captured on paper based registers: chronic cough register for presumptive TB cases, district TB register for active TB cases and TB patient cards for individual monitoring of TB treatment (Table 1). The central prisons have their own TB treatment registers (same as the district TB registers). All prisoners diagnosed with TB are also captured in the district TB treatment register so that the district can keep track of the prisoners. Data is collected on a quarterly basis by the National TB program through an integrated HIV/TB program supervision.

**Table 1:** Operational definitions of TB treatment outcomes

**Cured**	A pulmonary TB patient with bacteriologically confirmed TB at the beginning of treatment who was smear- or culture-negative in the last month of treatment and on at least one previous occasion.
**Treatment completed**	A TB patient who completed treatment without evidence of failure but with no record to show that sputum smear or culture results in the last month of treatment and on at least one previous occasion were negative, because tests were not done or because results are unavailable.
**Treatment success**	Sum of cured and treatment completed.
**Treatment failed**	TB patient whose sputum smear or culture is positive at month 5 or later during treatment**.**
**Died**	TB patient who dies for any reason before starting or during the course of treatment.
**Loss To Follow Up**	TB patient who did not start treatment or whose treatment was interrupted for 2 consecutive months or more.
**Not Evaluated**	TB patient for whom no treatment outcome is assigned. This includes cases “transferred out” to another treatment unit as well as cases for whom the treatment outcome is unknown to the reporting unit.

### Study participants

**Fig. 1:**
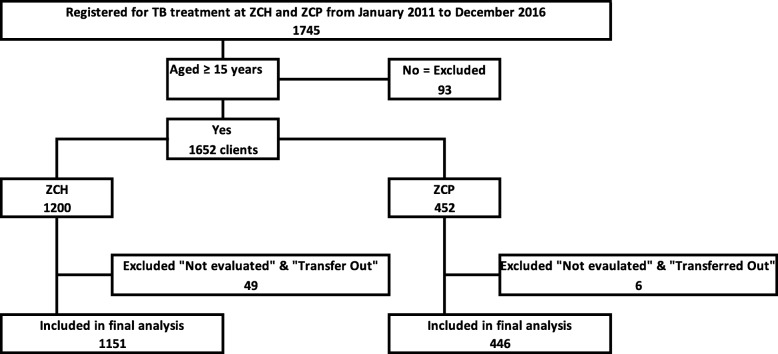
Registered TB cases at the Zomba Central Hospital and Zomba Central Prison from 2011 to 2016 in Zomba, Malawi

All prisoners and the general population aged 15 years and above who were diagnosed and put on TB treatment at the Zomba Central Prison and Zomba Central Hospital from January 2011 to December 2016 were included (Fig. 1).

### Ethical consideration

Ethical approval was obtained from the College of Medicine Research Ethics Committee (COMREC Reference No: P.05/18/2394). Individual informed consent was not sought since the study used fully anonymized retrospective data. Prior written authorization was obtained from Zomba Central Hospital, Zomba District Health Office and Malawi Prison Services.

### Data collection, management and analysis

We used routinely collected data extracted anonymously from the national standardized monitoring tools in ZCP and ZCH: TB register (age, sex, occupation, site of disease, history of previous treatment, type of diagnosis, treatment outcome, HIV test status, Anti –Retroviral Therapy {ART} and Cotrimoxazole Prophylactic Treatment {CPT} status). The data was collected in an anonymized way, entered into a data extraction sheet and then into the Access database then analyzed by STATA 13. Descriptive analysis was used to determine the patient characteristics and treatment outcomes. Chi squared tests were used to compare the characteristics between the prisoners and the general population. Bivariate logistic regression was used to assess associations between patient characteristics and poor treatment outcomes. The risk factors for poor TB treatment outcomes were analyzed using multivariate analyses adjusted for potential confounders. Odd ratios were provided with 95% confidence intervals and *p*-values (using a statistical significance level of < 0.05).

## Results

### Socio-demographic and clinical characteristics of the patients

Table 2 shows the baseline characteristics of the 1652 registered adult TB patients. Twenty seven percent (27%) were prisoners (all males) and 72% representeda general population (58% males). The median age was 35 years (IQR: 29–42); 76% were Pulmonary TB cases (78% among prisoners vs 75% among general population); 83% were new TB cases (77% among prisoners vs 86% among general population); and 65% were HIV positive (50% among prisoners vs 71% among general population).

**Table 2:** Socio-demographic and clinical characteristics of the patients

Characteristic	Total n (%)	Zomba Central Hospital, n (%)	Zomba Central Prison, n (%)
**Total participants**	1652	1200 (72.6)	452 (27.4)
***Sex (N = 1651)***			
Male	1148 (69.5)	697 (58.0)	451 (100.0)
Female	503 (30.5)	503 (42.0)	0 (0)
***Age(N = 1648)***			
Median (IQR)	35 (29.4)		
15–24	196 (11.9)	138 (11.5)	58 (12.8)
25–34	626 (38.0)	424 (35.3)	202 (44.7)
35–44	488 (29.6)	350 (29.2)	138 (30.5)
45–54	176 (10.7)	141 (12.0)	35 (8..0)
= > 55	162 (9.8)	145 (12.1)	17 (4.0)
***TB Registration Year(N = 1652)***			
2011	286 (17.1)	212 (17.7)	74 (16.3)
2012	302 (18.3)	246 (20.5)	56 (12.4)
2013	250 (15.1)	201 (16.8)	49 (10.8)
2014	270 (16.3)	193 (16.1)	77 (17.4)
2015	255 (15.4)	178 (14.8)	77 (17.4)
2016	289 (17.5)	170 (14.1)	119 (26.3)
***TB Form(N = 1652)***			
Pulmonary TB (bacteriologically, clinically)	1257 (76.1)	904 (75.3)	353 (78.1)
Extra-Pulmonary TB	395 (23.9)	296 (24.7)	99 (21.9)
***TB Category(N = 1652)***			
New	1376 (83.3)	1027 (85.5)	349 (77.2)
Relapse	79 (4.8)	46 (3.8)	33 (7.3)
Return After Loss To Follow Up	4 (0.2)	4 (0.3)	0 (0)
Failed	1 (0.1)	1 (0.1)	0 (0)
Other	191 (11.6)	122 (10.2)	70 (15.5)
***HIV Status(N = 1652)***			
Positive	1080 (65.4)	853 (71.1)	227 (50.2)
Negative	530 (32.1)	319 (26.6)	211 (46.7)
Unknown	42 (2.5)	28 (2.3)	14 (3.1)
***TB treatment outcomes(N = 1652)***			
Cured	479 (29.0)	308 (25.6)	171 (37.8)
Treatment Completed	993 (60.1)	743 (61.9)	250 (55.3)
Treatment Failed	2 (0.2)	2 (0.2)	0 (0)
Died	122 (8.1)	97 (8.0)	25 (5.5)
Transferred Out	12 (0.7)	12 (1.0)	0 (0)
Not Evaluated	43 (2.6)	37 (3.1)	6 (1.3)

*The percentage in the 5 year trend section is provided as a percentage of the total number over 6 years

### Trends in TB registrations and treatment outcomes

Overall all forms of TB were gradually decreasing among the prisoners from 2011 to 2013, and the trend increased from 2014 to 2016 while in the general population, all forms of TB peaked in 2012 and gradually declined from 2013 to 2016.There is a significant difference in the trend of all forms of TB among prisoners and the general population(X^2^ trend = 26.1: *P* < 0.05).

**Fig. 2:**
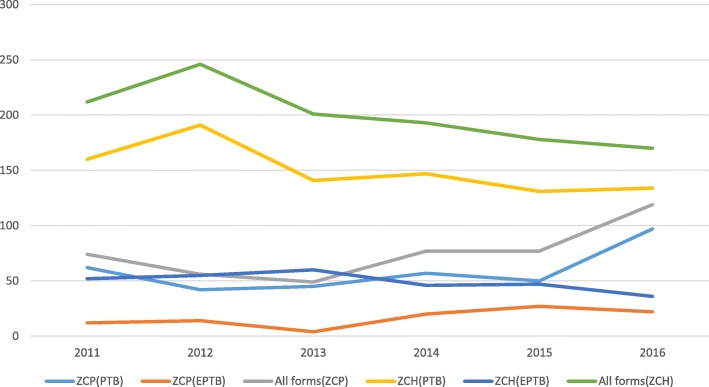
Trend of all forms registered TB cases at Zomba Central Prison and Zomba Central Hospital in Malawi. There is a significant difference in the trend of all forms of TB among the prisoners and the general population (X^2^ = 26.1; *P* < 0.05)

**Fig. 3:**
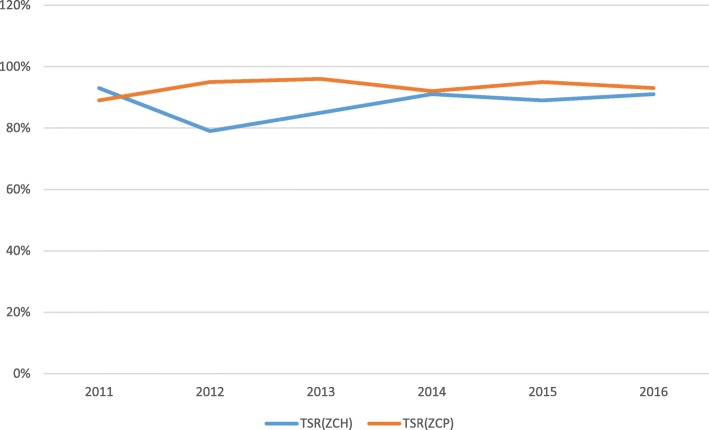
Trend of TB treatment success rates (TSR) among prisoners at Zomba Central Prison (ZCP) and the general population at Zomba Central Hospital (ZCH) in Malawi. There is a significant difference in the trend of all forms of TB among the prisoners and the general population (X^2^ = 33.2; P < 0.05)

Among the prisoners, the PTB cases plateaued from 2011 to 2015, and thetrend increased in 2016 while among the general population, the PTB cases peaked in 2012 and gradually declined from 2013 to 2016. EPTB cases in the two populations remained stable over the 6 year period (Fig. 2). There was a difference in the treatment outcomes among the two populations(X ^2^ = 33.2; P < 0.05). Overall, the mean TSR was 93% among prisoners and 88% in thegeneral population), 2(0.2%) were treatment failures (general population), 122 (8%) died (5% among prisoners vs 8% among general population) and 55 (3%) were not evaluated (1% among prisoners vs 4% among general population). In the general population, the trend for the TSR declined in 2012 and gradually increased from 2013 to 2016 while it remained fairly constant among the prisoners over the study period (Fig. 3 and Table 2).

### Factors associated with unsuccessful treatment outcomes

Multivariate logistic regression showed that age greater than 35 years (aOR = 0.68: 95% C.I: 0.58–0.80), Extra-Pulmonary TB (aOR = 1.69: 95% C.I: 1.08–2.63) and HIV positive status (aOR = 0.63: 95% C.I: 0.42–0.94) were the factors associated with unsuccessful TB treatment outcomes (Table 3).

**Table 3:** Factors associated with unsuccessful treatment outcomes (death and treatment failure), *n* = 1597

Variable	Total (%)	Treatment Success	Unsuccessful Treatment	Crude OR (95% CI)	P-value	Adjusted OR (95% CI)	P-value
**Sex**							
Female	486 (30.5)	444 (91.4)	42 (8.6)	1			
Male	1110 (69.5)	1027 (92.5)	83 (7.5)	1.17 (0.79–1.72)	0.43		
**Age**						**0.68 (0.58–0.80)**	**0.00**
15–24	186 (11.7)	180 (96.8)	6 (3.2)	1			
25–34	606 (38.0)	566 (93.4)	40 (6.6)	0.47 (0.20–1.13)	0.09		
35–44	472 (29.6)	436 (92.4)	36 (7.6)	**0.40 (0.17–0.97)**	**0.04**		
45–54	172 (10.8)	156 (90.7)	16 (9.3)	**0.32 (0.12–0.85)**	**0.02**		
= > 55	157 (9.9)	130 (82.8)	27 (17.2)	**0.16 (0.06–0.40)**	**0.00**		
**Facility**						1.06 (0.62–1.79)	0.82
Zomba Central Prison	446 (27.9)	421 (94.4)	25 (5.6)	1			
Zomba Central Hospital	1151 (72.1)	1051 (91.3)	100 (8.7)	**1.60 (1.01–2.51)**	**0.04**		
**TB Registration Year**						0.92 (0.52–1.38)	0.98
2011	279 (17.5)	263 (94.3)	16 (5.7)	1			
2012	276 (17.3)	247 (89.5)	29 (10.5)	0.53 (0.27–1.03)	0.06		
2013	241 (15.1)	217 (90.0)	24 (10.0)	0.51 (0.26–1.00)	0.05		
2014	269 (16.8)	247 (90.2)	22 (8.2)	0.66 (0.33–1.30)	0.23		
2015	249 (15.6)	232 (93.2)	17 (6.8)	0.90 (0.43–1.88)	0.78		
2016	283 (17.7)	266 (94.0)	17 (6.0)	0.91 (0.44–1.86)	0.79		
**TB Form**						**1.69 (1.08–2.63)**	**0.01**
Pulmonary TB	1214 (76.0)	1130 (93.1)	84 (6.9)	1			
Extra-Pulmonary TB	383 (24.0)	342 (89.3)	41 (10.7)	**1.61 (1.08–2.38)**	**0.02**		
**TB Category**						0.97 (0.67–1.40)	0.88
New	1332 (83.4)	1233 (92.6)	99 (7.4)	1			
Relapse/ Return After Loss To Follow Up/Failed	78 (4.9)	76 (97.4)	2 (2.6)	3.04 (0.73–12.59)	0.12		
Other	186 (11.7)	162 (87.0)	24 (13.0)	0.54 (0.33–0 .87)	**0.01**		
**HIV Status**						**0.63 (0.42–0.94)**	**0.02**
Negative	518 (32.4)	497 (96.0)	21 (4.0)	**1**			
Positive	1042 (65.3)	944 (90.6)	98 (9.4)	**0.40 (0.25–0.66)**	**0.00**		

## Discussion

In this 6 year retrospective study, the results show differences in the trend of all forms of TB and TB treatment success rates among the prisoners and the general population. Age greater than 35 years, HIV positive status and extra – Pulmonary TB were independently associated with unsuccessful TB treatment outcomes (death and treatment failure). In addition, it was found that the prison only registered male TB patients over the course of the 6 years.

There was an increase in the TB cases at the Zomba Central Prison from 2014 to2016 due to the introduction of the TB mass screening campaigns using a mobile X-ray machine while at the Zomba Central Hospital, the TB cases peaked in 2012 and thereafter there was a steady decline from 2013 to 2016. This might be attributed to the further scale up of antiretroviral therapy program through the Option B plus which was rolled out in 2011 in Malawi [12].

There were differences in the mean TB TSR among the two study populations (93% among prisoners and 88% among the general population. However, the 89% overall TB treatment Success Rate (TSR) is comparable to other studies done in resource limited settings and it is within reach of the End TB Strategy target of = > 90%. Similar studies conducted in the general population (from 2008 to 2010) at a Large District Hospital [8] and a Central Hospital [13] in Malawi and Ethiopia showed an 86% TSR ([14]. This is also higher than the 73% TSR [15] from 27 prisons in Malawi in 2007, 66% TSR reported in Zambian prison [16] in 2010–2011 and 48% TSR in Ugandan prisons [6] from 2011 to 2012. However, the TSR is comparable to recent studies done in prisons in sectionEthiopia [17] (89, 90%) but slightly lower than the study done in South Africa [5] (92%) and Ethiopia [17] (94%). The higher TSR in prison than the general population might be attributed to the maximum security prisons where the prisoners incarcerated for a longer sentences are bound to be within the prison and within reach of the wardens for the DOTS [16]. The varied intra prison differences may be due to the high turnover of prisoners andremandees (they are not yet sentenced and can be transferred out to another facility or released into the general public where they are lost to follow up) [16]. There can be poor linkage within prison facilities and the communities as once prisoners are released into the community, they are not adequately actively followed up for continuity of treatment [18, 19].

This studyshowed an overall 2.6% loss to follow-up which was lower than in other studies reported in similar limited resource setting in prisons in Uganda (43.0%) [6], and Brazil (13.0%) [4]. The lower rate of loss to follow up is attributed to the stable prison population at the Zomba Central Prison that is serving long term sentences hence not easily transferred or released [16].

The 5.5% death rate is comparable to the death rate observed in Uganda prison [6] but higher than the 1.4% death rate recorded in Ethiopia [17], 1.8% death rate recorded in South Africa [5] and 2% death rate recorded in Brazil [4]. This high death rate could be attributed to the poor prison living conditions -overcrowding [18], poor nutrition [19] and possibly to the rate of HIV/TB co-infection without use of antiviral therapy (due to the lower coverage of antiretroviral therapy use during this time period), which has been shown to be associated with unsuccessful treatment outcome [8, 20]. This study also showed that unsuccessful TB treatment outcome was associated with age greater than 35 years old. Similar studies in Botswana and Nigeria have shown that older age may be confounded by the high risk behaviorse.g. alcohol and drug use which are common in prison settings and general population especially among men. This leads to poor adherence which result to poor treatment outcomes.

A common risk factor for unsuccessful TB treatment outcome, Extra Pulmonary TB was also found in this study. This might be due to the severe nature of the EPTB,delays in diagnosing EPTB [21] duelimited diagnostic capacity and lack of treatment monitoring tests for EPTB cases [22].

In this study, gender comparisons were not possible among prisoners and the general population because the prison registered male TB patients only in the whole study period. Similar studies globally have shown unevenly distributed high proportions of male prisoners than female prisoners ([19, 23, 24]).Globally, there has been no universally accepted explanation to this disparity and most criminology studies point to the socialized gender roles and different expectationsof male and female behaviors [25].

The general findings from this study shows that prisoners can achieve good TB treatment outcomes which are comparable to the general population. The long serving prisoners in a maximum security prison provides an opportunity of uninterrupted treatment since they are not easily transferred out to other facilities. This calls for continued support to the prison health programs [26]. Several strategies can be combined to yield successful efforts in the fight against TB in the prison setting. TB mass screenings can be used to increase TBcase detection [24]. Since the TB treatment outcomes are worsened by HIV positive status [5, 8, 20] scaling up of the 3 phase integrated screening and treatment for HIV, TB and nutrition during entry, stay and exit of prison could increase access to HIV, TB and Nutritional screening and subsequent linkage to appropriate treatment services.

The strengths of this study include the following that we used routine program based data that is collected from the facility national TB registers which are used for TB patient registration, monitoring and evaluation from within a national public system. We studied a large number of TB patients and the results are representative of the national TB TSR. The study adhered to STROBE (Strengthening the Reporting of Observational Studies in Epidemiology) guidelines for the reporting of observational data.

Some noted limitationsinclude the use of retrospective data in which other important risk factors for unsuccessful TB treatment outcomes were not assessed (smoking status, alcohol status, body mass index, disaggregation of EPTB and HIV, and TB drug side effects). The analysis also excluded patients who were transferred out and those not evaluated which might posed a bias.

## Conclusion

Good TB treatment outcomes which are comparable to the general population were achieved among Malawian prisoners despite the challenging prison conditions due to the stable nature of the long serving prisoners in a maximum security prison who do not have TB treatment interruptions and health care workers’ and technical assistance support from Dignitas International. This calls for continued support for prison health programs. Further longitudinal research is required to look at the other contributing factors in details.

## Data Availability

The datasets used during the current study are available from the corresponding author on reasonable request.

## Abbreviations

ART: Antiretroviral therapy
COMREC: College of Medicine Research Ethics Committee
CPT: Cotrimoxale prevention therapy
DI: Dignitas International
DOTS: Direct observed treatment strategy
EPTB: Extra pulmonary tuberculosis
MDR-TB: Multi-drug resistant tuberculosis
TSR: Treatment success rate
TB: Tuberculosis
WHO: World Health Organization
ZCH: Zomba Central Hospital
ZCP: Zomba Central Prison

## References

[1] WORLD HEALTH ORGANIZATION. GLOBAL TUBERCULOSIS Report 2019. S.l.: WORLD HEALTH ORGANIZATION; 2019.

[2] Telisinghe L, Charalambous S, Topp SM, Herce ME, Hoffmann CJ, Barron P (2016). HIV and tuberculosis in prisons in sub-Saharan Africa. Lancet Lond Engl.

[3] Baussano I, Williams BG, Nunn P, Beggiato M, Fedeli U, Scano F. Tuberculosis Incidence in Prisons: A Systematic Review. PLoS Med 2010;7(12). Available from: https://www.ncbi.nlm.nih.gov/pmc/articles/PMC3006353/. [cited 2020 Apr 11]. 10.1371/journal.pmed.1000381 PMC3006353 21203587

[4] Ribeiro Macedo L, Reis-Santos B, Riley LW, Maciel EL (2013). Treatment outcomes of tuberculosis patients in Brazilian prisons: a polytomous regression analysis. Int J Tuberc Lung Dis Off J Int Union Tuberc Lung Dis.

[5] Mnisi T, Tumbo J, Govender I (2013). Factors associated with pulmonary tuberculosis outcomes among inmates in Potchefstroom prison in north west province. South Afr J Epidemiol Infect.

[6] Schwitters A, Kaggwa M, Omiel P, Nagadya G, Kisa N, Dalal S (2014). Tuberculosis incidence and treatment completion among Ugandan prison inmates. Int J Tuberc Lung Dis Off J Int Union Tuberc Lung Dis..

[7] Abouyannis M, Dacombe R, Dambe I, Mpunga J, Faragher B, Gausi F (2014). Drug resistance of mycobacterium tuberculosis in Malawi: a cross-sectional survey. Bull World Health Organ.

[8] Tweya H, Feldacker C, Phiri S, Ben-Smith A, Fenner L, Jahn A (2013). Comparison of treatment outcomes of new smear-positive pulmonary tuberculosis patients by HIV and antiretroviral status in a TB/HIV clinic, Malawi. Plos One.

[9] Ali SA, Mavundla TR, Fantu R, Awoke T (2016). Outcomes of TB treatment in HIV co-infected TB patients in Ethiopia: a cross-sectional analytic study. BMC Infect Dis.

[10] Sinshaw Y, Alemu S, Fekadu A, Gizachew M (2017). Successful TB treatment outcome and its associated factors among TB/HIV co-infected patients attending Gondar University referral hospital, Northwest Ethiopia: an institution based cross-sectional study. BMC Infect Dis.

[11] Nglazi MD, Bekker L-G, Wood R, Kaplan R. The impact of HIV status and antiretroviral treatment on TB treatment outcomes of new tuberculosis patients attending co-located TB and ART services in South Africa: a retrospective cohort study. BMC Infect Dis 201515. Available from: https://www.ncbi.nlm.nih.gov/pmc/articles/PMC4653912/. [cited 2018 Jan 4]. 10.1186/s12879-015-1275-3 PMC4653912 26584607

[12] Kanyerere H, Harries AD, Tayler-Smith K, Jahn A, Zachariah R, Chimbwandira FM (2016). The rise and fall of tuberculosis in Malawi: associations with HIV infection and antiretroviral therapy. Tropical Med Int Health.

[13] Kanyerere HS, Mpunga J, Tweya H, Edginton M, Harries AD, Hinderaker SG (2012). Timing of antiretroviral therapy and effects on tuberculosis treatment outcomes in HIV-co-infected patients in Malawi. Public Health Action.

[14] Berhe G, Enquselassie F, Aseffa A (2012). Treatment outcome of smear-positive pulmonary tuberculosis patients in Tigray region, Northern Ethiopia. BMC Public Health.

[15] Kanyerere HS, Banda RP, Gausi F, Salaniponi FM, Harries AD, Mpunga J (2012). Surveillance of tuberculosis in Malawian prisons. Public Health Action..

[16] Hatwiinda S, Topp SM, Siyambango M, Harris JB, Maggard KR, Chileshe C (2018). Poor continuity of care for TB diagnosis and treatment in Zambian prisons: a situation analysis. Tropical Med Int Health.

[17] Adane K, Spigt M, Dinant G-J (2018). Tuberculosis treatment outcome and predictors in northern Ethiopian prisons: a five-year retrospective analysis. BMC Pulm Med.

[18] Topp SM, Moonga CN, Luo N, Kaingu M, Chileshe C, Magwende G (2017). Mapping the Zambian prison health system: an analysis of key structural determinants. Glob Public Health.

[19] Topp SM, Moonga CN, Luo N, Kaingu M, Chileshe C, Magwende G (2016). Exploring the drivers of health and healthcare access in Zambian prisons: a health systems approach. Health Policy Plan.

[20] Kayigamba FR, Bakker MI, Mugisha V, Naeyer LD, Gasana M, Cobelens F (2013). Adherence to tuberculosis treatment, sputum smear conversion and mortality: a retrospective cohort study in 48 Rwandan clinics. PLoS One.

[21] Asres A, Jerene D, Deressa W (2018). Delays to treatment initiation is associated with tuberculosis treatment outcomes among patients on directly observed treatment short course in Southwest Ethiopia: a follow-up study. BMC Pulm Med.

[22] Ejeta E, Beyene G, Balay G, Bonsa Z, Abebe G (2018). Factors associated with unsuccessful treatment outcome in tuberculosis patients among refugees and their surrounding communities in Gambella regional state, Ethiopia. Plos One.

[23] Mitruka K, Volkmann T, Pratt RH, Kammerer JS (2017). Disparities in tuberculosis treatment completion by incarceration status, U.S., 1999–2011. Am J Prev Med.

[24] Morishita F, Garfin AMCG, Lew W, Oh KH, Yadav R-P, Reston JC, et al. Bringing state-of-the-art diagnostics to vulnerable populations: The use of a mobile screening unit in active case finding for tuberculosis in Palawan, the Philippines. PLoS One. 2017;12(2). Available from: https://www.ncbi.nlm.nih.gov/pmc/articles/PMC5289556/. [cited 2019 Oct 18]. 10.1371/journal.pone.0171310 PMC5289556 28152082

[25] Wehrman MM (2011). Examining race and sex inequality in recidivism. Sociol Compass.

[26] Dara M, Chadha SS, Melchers NV, van den Hombergh J, Gurbanova E, Al-Darraji H (2013). Time to act to prevent and control tuberculosis among inmates [official statement of the International Union against Tuberculosis and Lung Disease]. Int J Tuberc Lung Dis.

